# A mobile-based educational intervention on media health literacy: A quasi-experimental study

**DOI:** 10.34172/hpp.2023.28

**Published:** 2023-09-11

**Authors:** Mahsa Nazarnia, Fatemeh Zarei, Nasrin Roozbahani

**Affiliations:** ^1^Department of Health Education and Health Promotion, Faculty of Medical Sciences, Tarbiat Modares University, Tehran, Iran; ^2^Department of Health Education and Health Promotion, Faculty of Health, Arak University of Medical Sciences, Arak, Iran

**Keywords:** Health education, Information literacy, Health

## Abstract

**Background:**

Health misinformation on social media is a persistent public health concern that requires the proper skill set for interpreting and evaluating accurate information. This study aimed to determine the effects of a mobile app-based educational intervention on media health literacy (MHL) development among Iranian adults.

**Methods:**

This study was a quasi-experimental design conducted in 2022 that included 100 Iranian adults aged 18 to 65 years old. The inclusion criteria for participation were ownership of an Android smartphone, no prior training in MHL, and membership in at least one virtual social media app. As the primary outcome of the study, MHL was assessed using the validated MeHLit questionnaire with two follow-up time points (immediately after program completion and 12 weeks after program completion) in addition to a pre-test. Participants were divided into an experimental group that received the mobile app-based training program and a control group that received none.

**Results:**

The repeated measures test revealed a significant effect of the group-time interaction on the mean MHL score in both the intervention and control groups. Within the intervention group, the follow-up test indicated a significant increase in mean MHL scores for post-test 1 (63.54±12.57) and post-test 2 (65.72±7.97) compared to the pre-test phase (55.14±12.04), with these increases being statistically significant (*P*<0.001). No significant difference was observed within the control group.

**Conclusion:**

The results of this study suggest that the mobile app-based educational intervention was effective in improving MHL among Iranian adults. These findings highlight the potential of mobile app-based interventions for promoting MHL and addressing health misinformation on social media.

## Introduction

 In contemporary society, media’s educational influence is undeniable, and media consumption is unavoidable.^1^ Its impact on people’s health-related attitudes, beliefs, and actions is substantial, underscoring the need for scrutinizing transmitted content, especially health-related information. A crucial skill in the 21st century is media literacy,^2,3^ encompassing the capacity to access, analyze, and craft messages across diverse contexts.^4,5^ A media-literate individual engages critically with both consuming and producing media, decoding, evaluating, and creating various forms of media across mediums like radio, TV, internet, and more. Moreover, they recognize media’s influence on individuals and communities.^6^ European Media Literacy Index 2021^7^ highlights how media-literate individuals master skills in accessing, analyzing, and creating messages across contexts. Divergent Media Literacy Index scores in 2021 reveal higher media literacy (ML) in European nations like Finland, Denmark, etc., while certain Asian nations such as Iran^8-11^ and Indonesia^8,12^ exhibit lower scores. Media literacy is a predictive facet of health literacy,^11,13^ yielding positive effects on healthy lifestyles,^10,14^ distinguishing accurate information in social networks,^15^ and managing rumors and infodemics.^16,17^ Consequently, media literacy simplifies tasks like sourcing authentic health information,^18,19^ identifying symptoms,^20^ preventing behaviors like smoking,^21^ and promoting sexual health awareness and education.^22-26^

 Media health literacy (MHL)^27^ is the amalgamation of media literacy and health literacy, which considers the significance of digital media and health knowledge. It encompasses both locating health-related content in media and discerning implicit and explicit media content’s impact on health.^28^ False medical advice propagated by commercial entities and the healthcare system has been shown to drive the dissemination of misinformation about diseases, with virtual social media playing a key role.^10,29^ Mass media and societal elites wield substantial influence on socialization, attitudes, and health-related behaviors. While commonly assumed that doctors and healthcare providers would benefit from enhanced media literacy, health communication theories suggest otherwise.^30^ The study’s focus is on adults due to their multifaceted roles, such as parenting and decision-making, which are influenced by media consumption.^31,32^ This underscores the necessity of MHL skills to comprehend the formation of accurate and incorrect health beliefs on media platforms. By acquiring these skills, individuals can navigate the complex health information landscape across various media platforms and make well-informed health choices.^27^

 MHL, a unique concept, combines health literacy and media literacy, essential for personal development as per the Ottawa Charter for Health Promotion’s “Developing Personal Skills” action area.^33^ Education is pivotal to this development, requiring precise methods for intervention. Amidst the COVID-19 pandemic, online education has emerged as an effective means, allowing continuity while adhering to social distancing. Effective educational interventions for MHL encompass interactive online courses, health apps, and social media campaigns. The Media Wise program aids young people in identifying and combating misinformation, while the Health Navigator app provides personalized health information via mobile phones.^34^ Social media initiatives, like #chatsafe, involve youth recommendations for suicide prevention.^35,36^ Overall, diverse digital tools and platforms are leveraged for effective educational interventions in developing MHL skills.

 The importance of mHealth learning lies in its role in personal development and health promotion, particularly vital during the COVID-19 pandemic.^37-40^ Electronic and online education, offering flexibility, accessibility, and cost-effectiveness, is well-suited for delivering MHL education. Leveraging mobile phone-based health education programs effectively reaches intended audiences and delivers educational interventions. Successful outcomes in developing MHL skills hinge on selecting the right educational intervention approach. Therefore, this study evaluated the effects of a mobile-based educational intervention to promote MHL among Iranian adults. Two main research assumptions were defined as fallow: MHL and its domains were observed between the control and experimental groups. Therefore, two main research assumptions were defined as fallow:

1. A mobile-based educational intervention effect on MHL in the two intervention and control groups in three main phases; pre-test, and two post-tests.
2. A mobile-based educational intervention effect on five domains of MHL in the two intervention and control groups in three main phases; pre-test, and two post-tests.

## Materials and Methods

###  Study design and population

 A quasi-experimental study was conducted to evaluate the effectiveness of a mobile-based educational intervention on MHL among Iranian adults in 2022. The participants in this study were adults aged 18–65 years. The inclusion criteria of eligible participants were: (1) Iranian adults who owned an Android-based smartphone and were able to use it; (2) had not received prior MHL training; (3) were members of at least one virtual social media app; (4) willing to participate in this study with an informed consent form. Participants unwilling to continue were excluded from the study.

###  Sampling 

 Based on the study by Bahramian et al^41^ considering the mean score of one of the media literacy domains before (14.2) and after intervention (16.25), the standard deviation before (3.60) and after (2.57) the intervention, one error type (α = 0.05), and a power of 95% (β = 0.05) using Pocock’s formula, the sample size for each group was calculated to equal to 39 by G-Power software. If the lost follow-up rate of participants was 30%, the sample size in each group was modified equal to 50 in each group.

**Figure 1:**
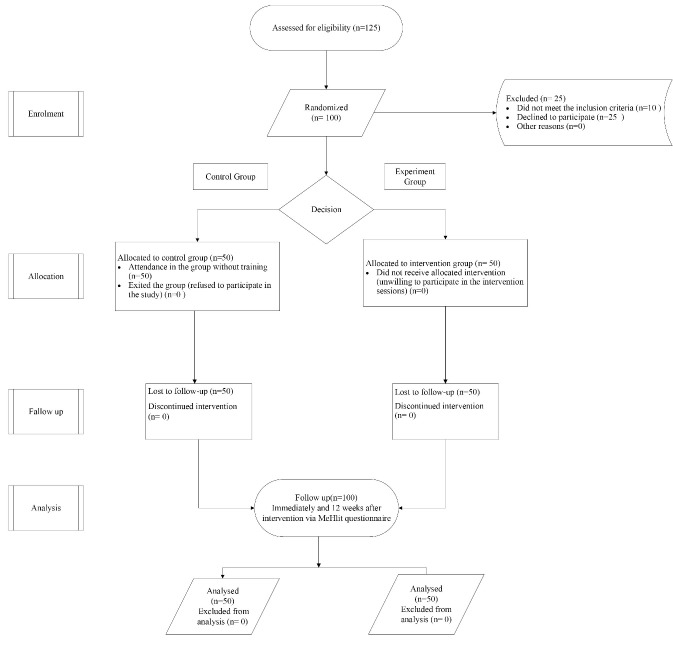
CONSORT flow diagram

 Convenience sampling started with an online announcement to participate in the study through social media apps such as Telegram, WhatsApp, Instagram, etc. In the announcement, along with the inclusion criteria, the purpose of the study and the research team’s contact number was included. The participation deadline was 10 days after the announcement was released. At the time of recruitment, a trained assistant who was not on the research team (SHH) started the sampling process. The purpose of the study was explained to the eligible and interested people, and they were assured that their information would remain completely confidential. The informed consent form was designed electronically and sent to the eligible individuals through social media apps and email. After releasing the online announcement, 532 people accepted the invitation link. Initially, 125 participants were assessed for eligibility through phone calls. Of the 25 participants who were excluded, 10 did not meet the inclusion criteria (participants had iOS phones and could not use the Android-based app), and 15 declined to participate. One Hundred participants were enrolled into two parallel groups; 50 were assigned to the experiment group (SORS-app group) and 50 were assigned to the control group. The qualified candidates were then listed along with their names, contact numbers, and emails. People were allocated into the two experiment and control groups by lottery. The study used simple randomization to ensure equal representation of participants in both the experimental and control groups, with adjustments made for any improvements in requirements. Randomization was carried out online through an Interactive Web Response System at the time of enrollment. To ensure allocation concealment, the randomization lists were kept in the possession of one of the investigators (SHH) who was not involved in the study intervention. The process of randomization and allocation of samples is illustrated in the CONSORT flow diagram (Figure 1).

###  Data collation and measurement

 The assessment tool was a standard questionnaire (MeHLit) designed specifically for MHL assessment.^42^ The acronym MeHLit stands for Media Health Literacy Tool and includes five domains; 1- Goal appraisal skill (7 items), 2- Content appraisal skill (5 items), 3- Implicit meaning appraisal skill (4 items), 4- Visual Comprehension skill (3 items), and 5- Audience appraisal skill (2 items). The MeHLit impact score was above 1.5. The reliability of the MeHLit questionnaire was evaluated using Cronbach’s alpha. The overall Cronbach’s alpha for MeHLit was found to be 0.91. The Cronbach’s alpha values for the five domains of the questionnaire were as follows: Goal Appraisal Skill (0.78), Content Appraisal Skill (0.82), Implicit Meaning Appraisal Skill (0.70), Visual Comprehension Skill (0.77), and Audience Appraisal Skill (0.70). All of the domains were found to be positively and significantly correlated with one another.

###  Development and implementation of the training program

 The intervention conducted between 12 January 2021 and 1 May 2022. Before initiating the study, 30 eligible participants were contacted via phone to determine their preferred platform for receiving educational materials. Participants were asked to choose between a mobile application or a website. Of the 30 participants surveyed, 25 expressed a preference for receiving information via the mobile application. An educational module for the self-learning component which consisted of a smartphone application called SORS was developed. This application was designed to provide individuals with a user-friendly tool for enhancing their MHL skills. This part of the study was designed in several following steps:

####  Step 1: Preparation and assessment of the educational contents for the SORS -app 

 In order to develop content for the SORS app, a thorough review of the literature on MHL and its key concepts, dimensions, critical thinking, internet addiction, fake news, infodemics, misinformation, and rumors was conducted. The materials were then reviewed by the research team who simplified and summarized the content for better understanding and turned them into messages, stories, movies, motion graphics, and educational recommendations (Table 1).

**Table 1:** Media health literacy domains, educational objectives, educational content, and content presentation format of the application

**MHL domains**	**Learning domain**	**Educational objectives**	**Topics**	**Presentation format**
Goal appraisal skill	Cognitive	The learner can acquire the skill of evaluating the purpose of the message in the media.	Fake News in Health	Long-winded text
			Media and Children’s Health	Long-winded text
	Emotional	The learner can pay attention to the purpose of the message when dealing with health-related messages.	Media advertisements for health	Video
	Psychomotor	The learner can use the skill of evaluating the purpose of the message when dealing with health-related messages.	Media advertisements for health	Video
Content appraisal skill	Cognitive & Emotional	The learner can acquire the skill of evaluating message content.	Media Consumption habit and Health	Long-winded text
			Infodemic/Fake news/ pseudoscience	Long-winded text
	Cognitive	The learner can pay attention to the skill of evaluating message content when dealing with health-related messages.	Media advertisements for health	Video
				Game
	Psychomotor	The learner can use the skill of evaluating message content when dealing with health-related messages.	Critical thinking	Video
Implicit meaning appraisal skill	Cognitive	The learner acquires the skill of evaluating the implicit meaning of the message.	Internet addiction/mobile addiction	Long-winded text
				Glossary
	Emotional	The learner can pay attention to the skill of evaluating the implicit meaning of the message when dealing with health-related messages.	Media and Culture	Video
			Media and mental health	Video
	Cognitive	The learner will be able to use the skill of evaluating implicit meaning when dealing with health-related messages.	The concept of News gatekeeper	Video
Visual comprehension skill	Cognitive	The learner can find the role of visual technique in the understanding of a message.	Role of colors in health	Game
			Role of Symbols in Health	Game
	Emotional	The learner can pay attention to the lights, colors, sounds, and special effects when dealing with health-related messages.	Media advertisements for health	Video
	Psychomotor	The learner can use the skill of visual understanding of the message when dealing with health-related messages.	Media and Culture	Video
Audience appraisal skill	Cognitive	The learner can define audience evaluation skills.	Media literacy principles	Long text
	Emotional	The learner can pay attention to the evaluation of the audience when dealing with health-related messages.		Video
Media literacy	Cognitive Emotional	The general meaning of media literacy can define.	Media consumption habits	Long-winded text
				Long-winded text
			Fake News in Health	Long-winded text
			Nomophobia	Long-winded text
	Cognitive	The learner can encourage others to acquire media literacy.	Media and obesity	Video
	Psychomotor	The learner can use media literacy when dealing with health messages.	Social media and health	Video

####  Step 2: Designing the mobile application 

 This step is followed by two sub-sections:

####  a. Designing the user interface and user experience of the SORS-app

 One of the most important skills a person with a good level of MHL acquires is to distinguish content from *reliable sources*. Hence, we coined the name ‘SORS’ for the application, which is an acronym derived from the initial letters of three Persian words: ‘Sawad’ meaning ‘literacy,’ ‘Resaneh for ‘media,’ and ‘Salamat’ for ‘health’. In addition, the pronunciation of SORS in Persian is the same as the word ‘SOURCE’ in English. Two colors were used in the design of the SORS app logo; white as a symbol of *Awareness*, and green as a symbol of *Health*. The SORS app is an Android-based application in Persian written in the JAVA programming language. A team of app designers created the application’s technical structure, user interface, and user experience.^43^

####  b. The SORS-app usability 

 The SORS app was evaluated with the usability test.^44^ First, several individuals who were not under study but had the same characteristics as the research participants were asked to install the SORS app on their phones. They were then asked about the application’s efficiency, page colors, icon placements, speed of access to content, order of content placement, and cultural-ethical considerations. These questions were asked verbally by telephone. After a thorough review by the research team, the collected comments were sent to the app design team. The corrections were made and the app was given to 20 people. After receiving partial satisfaction from these users, the final version of the application was released and prepared for the educational intervention. Currently, the app is available on Cafe Bazaar, which is the most famous online Android app store in Iran. The SORS app version 1.0.0 can be downloaded via the following link: https://cafebazaar.ir/app/sors.ir

####  Step 3: Procedures - pre-test stage, educational stage and post-test stage

 All participants with the inclusion criteria were explained the objectives of the study by the first author (MN). She was experienced in social marketing with more than 5 years of job experience as a freelancer. Participants were explained how the app functioned.

####  Pre-test stage

 The pre-test was done via the aforementioned questionnaire in an online format which was sent by social media (WhatsApp) to participants. The link to the questionnaire was sent to the participants individually through either WhatsApp or email. The participants were asked to provide their feedback to complete the questionnaires.

####  Educational stage

 After completion of the pre-test, the first author (MN) provided the experiment group the link to download the SORS app. The experiment group could access the educational content on MHL through the SORS app at specific intervals throughout the 4-week training period. Details on how to install and use the app were sent to each participant separately in the form of a tutorial video via WhatsApp. To ensure the accuracy of the SORS app installation, the participants were asked to send a screenshot of the installed application. The experiment group was trained in the SORS app for 4 weeks. During this training period, the experiment group received illustrated images that showed the importance of MHL every day at 10:00 AM. The participants could contact the researchers by either calling, messaging through social networks, or sending an email to sorsapplic@gmail.com.

####  Post-test stage

 The first post-test was done for both the intervention and control groups immediately after the training period was completed. At the end of the first post-test, SORS-app users in the experiment group received a notification from the first researcher (SSE) through WhatsApp that reminded them to complete the questions in another post-test 12 weeks later. The control group completed the questionnaire immediately after the experimental group’s educational intervention and again 12 weeks later. Both groups completed the same number of questionnaires. The control group received no educational content during the training program and post-tests but was provided with a link to download the SORS app at the end of the program.

###  Data analysis 

 The data underwent analysis through SPSS 16 software, involving assessments of homogeneity between the two groups using chi-square tests, Fisher’s exact test, and independent t-tests to evaluate demographic characteristics. For the dependent variables, measurements were conducted at baseline, immediately after the intervention, and again 12 weeks later, employing repeated-measures ANOVA. The assumption of covariance matrix sphericity was evaluated using Mauchly’s test. Based on its results, *P* values were reported with consideration for the Greenhouse-Geiser correction when applicable. Furthermore, to pinpoint specific changes and distinctions, a Tukey post-hoc test was carried out.

## Results

###  Participant characteristics

Table 2 shows that the characteristics of the experiment and control group participants were mostly similar. Most participants had a mean age of 35.02 (SD 8.96) and 37.72 (SD 6.98) years in the experiment and control groups, respectively. In both groups, the majority of the subjects were city-dwelling employees (Table 2).

**Table 2:** Demographic characteristics of the intervention and control group participants

**Demographic characteristics**		**Control**	**Intervention**	**Test results**
Age	Mean ± SD	37.72 ± 6.98	35.02 ± 8.96	*P* = 0.120
Education level, No. (%)	High school	5 (10)	7 (14)	*P* = 0.247
	Diploma	13 (26)	7 (14)	
	College Education	26 (52)	27 (54)	
	Graduate	6 (12)	9 (18)	
Gender, No. (%)	Female	40 (80)	44 (88)	*P* = 0.569
	Male	10 (20)	6 (12)	
Job-status, No. (%)	Unemployed	18 (36)	20 (40)	*P* = 0.569
	Employee	26 (52)	23 (46)	
	Freelancer	3 (6)	4 (8)	
	Retired	3 (6)	3 (6)	
Marital status, No. (%)	Single	13 (26)	11 (22)	*P* = 0.64
	Married	37 (74)	39 (78)	
Location, No. (%)	Urban	43 (86)	46 (92)	*P* = 0.338
	Rural	7 (14)	4 (8)	

###  Comparison of the mean MHL domain scores between the experiment and control group participants

 The mean MHL domain scores were compared between the experiment and control group participants (Table 3).

**Table 3:** Comparison of Media Health Literacy and its domains in the two intervention and control groups before, immediately after, and 12 weeks after the completion of educational intervention

**Variable**	**Score (mean SD)**						**Interaction effect:** ** Time Group**	**Time**	**Group **
	**Baseline**		**Immediate**		**12 weeks follow-up**				
	**Intervention**	**Control**	**Intervention**	**Control**	**Intervention**	**Control**			
Goal appraisal skill	17.06 ± 5.01	16.88 ± 6.48	20.16 ± 4.91	17.54 ± 5.32	22.10 ± 3.12	18.98 ± 4.06	*P* = 0.001	*P* = 0.000	*P* = 0.006
Content appraisal skill	13.52 ± 2.78	14.28 ± 3.78	15.02 ± 3.40	12.98 ± 3.92	14.90 ± 2.69	13.58 ± 3.35	*P* = 0.006	*P* = 0.363	*P* = 0.117
Implicit meaning appraisal skill	11.16 ± 2.93	11.22 ± 3.45	12.92 ± 2.42	10.18 ± 3.14	13.02 ± 2.19	11.44 ± 2.89	*P* = 0.049	*P* = 0.013	*P* = 0.001
Audience appraisal skill	6.22 ± 1.23	6.36 ± 1.57	6.62 ± 1.23	5.20 ± 2.15	6.46 ± 1.95	5.40 ± 1.94	*P* = 0.009	*P* = 0.112	*P* = 0.001
Visual Comprehension skill	7.18 ± 2.24	7.72 ± 2.57	8.82 ± 2.10	7.50 ± 3.00	9.24 ± 1.76	7.32 ± 2.23	*P* = 0.000	*P* = 0.008	*P* = 0.015
Media Health Literacy	55.14 ± 12.04	56.46 ± 16.93	63.54 ± 12.57	58.47 ± 14.22	65.72 ± 7.97	56.72 ± 9.42	*P* = 0.000	*P* = 0.001	*P *= 0.003

####  Goal appraisal skill

 The repeated measures test showed that group-time interaction significantly impacted the mean goal appraisal skill scores of the intervention and control groups. The follow-up test in the intervention group showed that the mean post-test 1 and post-test 2 scores increased significantly when compared with the pre-test score (*P* < 0.001). Furthermore, the post-test 2 goal appraisal skill score increased significantly when compared with the post-test 1 score (*P* = 0.014). In the control group, the difference between the mean goal appraisal skill scores of the pre-test and post-test 1 stages was not significant, whereas the post-test 2 scores showed a significant increase over the obtained pre-test score (*P* = 0.031). Moreover, there was no significant difference between the goal appraisal skill scores of the post-test 1 and post-test 2-time points.

####  Content appraisal skill

 The repeated measures test showed that group-time interaction significantly affected the mean content appraisal skill scores of both groups under study (*P* < 0.006). The follow-up test in the intervention group showed that when compared with the pre-test stage, the mean content appraisal skill scores in the post-test 1 and post-test 2 stages had increased significantly (*P* < 0.01). There was no significant difference between the content appraisal skill scores of the post-test 1 and post-test 2-time points. In the control group, no statistically significant difference was observed between the different periods.

####  Implicit meaning appraisal skill

 The repeated measures test showed that group-time interaction had a significant effect on the mean implicit meaning appraisal skill scores of the intervention and control groups (*P* < 0.049). The follow-up test in the intervention group, when compared with the pre-test period, revealed a statistically significant increase in the mean implicit meaning appraisal skill score (*P* < 0.01). However, the mean implicit meaning appraisal skill scores of the post-test 1 and post-test 2-time points were not significantly different. In the control group, the mean implicit meaning appraisal skill score of the post-test 1 stage was significantly less than the pre-test value. However, the difference between the pre-test and post-test 2 mean scores was not statistically significantly, a significant difference was observed between the post-test 1 and post-test 2 mean scores.

####  Visual comprehension skill 

 The repeated measures test showed that group-time interaction significantly affected the mean visual comprehension skill scores of the intervention and control groups (*P* < 0.001). The follow-up test in the intervention group showed that when compared with the pre-test stage, the mean visual comprehension skill scores in the post-test 1 and post-test 2-time points had increased significantly (*P* < 0.001); However, there was no significant difference between the mean visual comprehension skill scores of the two post-tests. In the control group, the mean visual comprehension skill scores of the study’s three-time points were not different.

####  Audience appraisal skill

 The repeated measures test showed that group-time interaction significantly impacted the mean audience appraisal skill scores of both study groups (*P* < 0.009). The follow-up test in the intervention group showed that the mean audience appraisal skill score did not significantly change at different time points. In the control group, the mean audience appraisal skill scores of the post-test 1 and post-test 2-time points revealed a statistically significant decline, when compared with the pre-test value (*P* < 0.01). However, there was no difference between the post-test 1 and post-test 2 results.

####  Media health literacy

 The repeated measures test showed that the group-time interaction had a significant effect on the mean MHL score in the intervention and control groups. The follow-up test in the intervention group showed that when compared with the pre-test phase, the mean post-test 1 and post-test 2 MHL scores increased significantly (*P* < 0.001). However, the difference between the post-test 1 and post-test 2 mean MHL scores was not significant. No significant difference was observed in the control group.

## Discussion

 The study aimed to evaluate the effects of a mobile-based educational intervention on MHL among Iranian adults. The results support the hypothesis that educational interventions can increase MHL. The study found that the mean score of MHL significantly increased after four weeks of training through the SORS app, indicating the sustainability of the training program.

 Several studies have emphasized the effectiveness of MHL in public health fields, such as sexual health in adolescents,^45^ substance abuse prevention,^46^ sexual health education,^22,47^ and media literacy education for teachers.^48^ The results of all these studies are consistent with those of the present study. The results of a study by Scull et al were also in line with our results; in a clinical trial, they showed that web-based media literacy training had a positive effect on adolescents’ sexual health education.^2^ Moreover, in the study by Austin et al, a 6-session media literacy training program empowered parents and youth in critical thinking, and reduced the effects of food marketing on their families. They learned how to use the media to obtain correct nutritional information.^49^ These findings confirm the results of our study. To evaluate the sustainability of the training program, we conducted two follow-ups; immediately and three months post-intervention. Similarly, in the study by Solhi et al in 2017, the experimental group underwent two literacy training sessions. The subjects were followed up one- and three-month post-intervention and reported a positive attitude toward the correct use of weight loss supplements.^50^

 According to available evidence, MHL is an effective approach to health promotion,^20^ and media literacy training is a new strategy that is applied to reduce the harmful effects of social media messages in various areas of public health.^51-54^ Compared to the results of a cross-sectional descriptive study by Jones-Jang et al, information literacy alone (media, information, news, and, digital literacy) significantly increased the probability of identifying fake news. It was contrary to the results of our study.^55^ This discrepancy could be because the Jones-Jang and colleagues’ study was descriptive, and the target group was teenagers. Moreover, Jones-Jang and colleagues’ research referred to political ideologies. But the current project was a quasi-experimental study that focused on health-related issues among adults.

 The results showed that the mean goal appraisal skill score increased significantly at the post-test 1 and post-test 2-time points, which indicates the sustainable impact of the intervention. In 2021, Hwang et al conducted a pilot study to investigate the negative impact of misinformation, including deep-fakes, and the protective effect of media literacy education. The results of their study showed that media literacy training reduced the effects of disinformation, including deep-fakes, and caused participants to pay more attention when faced with deep-fakes. This suggests that media literacy education can be an effective tool for helping individuals identify and critically evaluate disinformation, including deep-fakes.^56^ It should be noted that Hwang and colleagues’ study was conducted on the web platform, which is relatively less productive when compared with the current study (through a mobile app) because the SORS app was always available to the experimental group provided strong evaluation.

 According to the results of the present study, the mean content appraisal skill score increased significantly at the post-test 1 and post-test 2-time points, which indicates the sustainable impact of the intervention. Education plays a fundamental role in developing the ability to understand the content of communication.^57^ In 2018, Bahramian et al carried out a quasi-experimental study entitled “Effect of educational intervention on media literacy of female high school students” over the course of 7 training sessions. The results showed that media literacy training can be practically included in schools as a strategy to improve the critical appraisal of produced media content and promote its goal-oriented and selective use. These findings were consistent with our results.^41^ A study by Pinkleton et al evaluated media content based on the message interpretation process model and found that participants who received media literacy training had a better understanding of how the media influences adolescent decision-making about sexual issues. This suggests that media literacy training can be an effective tool for helping young people make informed decisions about their sexual health.^58^ In our view, the most compelling explanation for the present set of findings is that media literacy training will enable society to cognitively and emotionally evaluate useless media content, avoid fake news, and filter out what they do not need.

 In the present study, the mean implicit meaning appraisal skill scores of the post-test 1 and post-test 2-time points significantly increased, when compared with the pre-test stage. In a similar study,^41^ the educational intervention increased the “*Viewpoints are displayed in the media message or removed from it”* domain score after 8 weeks of training, which was in accordance with the implicit meaning appraisal skill in the present study.

 Our findings highlighted the fact that the SORS app intervention improved the visual comprehension of adults. In the intervention group, the post-test 1 and post-test 2 visual comprehension skill scores increased significantly when compared with the pre-test scores. This result revealed the training program’s sustainability about this domain. By evaluating the authenticity of a message’s visual elements, a user can critically analyze any visual manipulation present in the message.^48^ A study conducted by Golding and Verrier found that training in visual literacy can enhance comprehension of educational comics.^59^

 Furthermore, Lopatovska et al conducted a visual literacy intervention using artistic images. The results showed that children’s knowledge of visual elements improved after training, which was consistent with the results of the present study.^60^ Results of previous study show that consumers in high-context communication systems are more able to derive implicit meaning from visual images in print ads than consumers in low-context communication systems.^61^ It seems that the role of culture in the understanding of visual concepts in the field of health can be considered and its expansion needs to be investigated.

 In the end, when compared with the pre-test results, the mean audience appraisal skill scores of the post-test 1 and post-test 2-time points were not significantly different. This meant that the intervention was not effective in improving this skill. The reason behind this was the lack of relevant educational content. Study of Bahramian et al in 2018 showed that 7 face-to-face training sessions improved the “media impact on society and individuals” and “preference in the media use” domain scores, which contradicts the results of our study.^41^

## Strengths and limitations

 Limitations of the study include the use of a quasi-experimental design, which limits the ability to establish causality and rule out alternative explanations for the results. Additionally, the educational content only provided on the Android-based app had to be excluded from the study for participants with iOS mobile phones, which lengthened the sampling time. Future research could address these limitations by using dual designs and recruiting participants from multiple operating systems. knowing the frequency of app use among the participants could be beneficial to assess the intention of participants about the developed content. The SORS-App has not had this feature. Additionally, further investigation is needed to determine whether the improvements in MHL seen in this study translate to improved health outcomes in real-world settings. This study represents an initial effort to combine two fields of research, Media Literacy and Health, which have not been directly connected before, to our knowledge. While the general applicability of the findings must be confirmed by future studies, the current research provides strong evidence in favor of educational interventions for MHL. Using a proper training method will be necessary for effectiveness. According to the findings of this research, the use of mobile phone applications as a tool for education can be useful along with other conventional educational methods. Furthermore, the use of these tools is much more economical, especially during pandemics when conducting face-to-face training courses is limited. Based on the studies on the role of healthcare workers in the production of health content and the need for them to be aware of media literacy, it may have been better if the healthcare workers were selected as the samples of this study. However, involving them in research may have increased their workload during the third wave of the COVID-19 in Iran. Therefore, the authors of this study strongly suggest future researchers design and implement an educational program to promote media literacy among healthcare workers. Overall, the study highlights the potential of technology-based interventions to improve health literacy and calls for continued efforts to promote health literacy in the population.

## Implication for Practice

 The findings of this study have important implications for public health practice. The demonstrated effectiveness of the mobile app-based educational intervention in improving MHL among Iranian adults suggests that similar interventions could be used to promote MHL and address health misinformation on social media. Health professionals and organizations could consider incorporating mobile app-based interventions into their health promotion and education efforts to improve MHL among the general population.

## Conclusion

 The research results indicated a significant difference in media literacy and its domains between the control and experimental groups at the two post-test time points. The largest mean differences between the control and experimental groups were observed in the two-goal appraisal and content appraisal skill domains. Apparently, to obtain the appropriate information in virtual space, these two skills are crucial, and a person equipped with these skills may be protected from misinformation, fake news, and even infodemic consequences. Although the implicit meaning appraisal, visual comprehension, and audience appraisal skills differed significantly in the two groups under study, the first two skills were undoubtedly more important for the content receiver.

## Acknowledgements

 We would like to extend our sincere gratitude to all participants in this study. Furthermore, we appreciate the support of the Vice Chancellor of Research in Tarbiat Modares University’s Faculty of Medical Sciences in Tehran, Iran.

## Competing Interests

 The authors declare no conflict of interest.

## Ethical Approval

 This study was conducted in compliance with all relevant ethical guidelines and regulations, including the Helsinki Declaration of Ethical Principles for Medical Research. The Ethics Committee of the Faculty of Medical Sciences at Tarbiat Modares University (IR.MODARES.REC.1400.234) granted ethical approval for the study. Data was collected through an online questionnaire, and informed consent was obtained from all eligible participants who volunteered to take part in the study.

## Funding

 This study was supported by the Faculty of Medical Sciences at Tarbiat Modares University in Tehran, Iran. This research received no external funding.

## Supplementary Files


Supplementary file 1 contains sections of the SORS-App.
