# Supplementary figures and images for: A mobile-based educational intervention on media health literacy: A quasi-experimental study
Source: Health Promot Perspect. 2023 Sep 11;13(3):227–36. doi: 10.34172/hpp.2023.28 (PMC10558972; doi:10.34172/hpp.2023.28)

Supplementary file 1: contains sections of the SORS-App

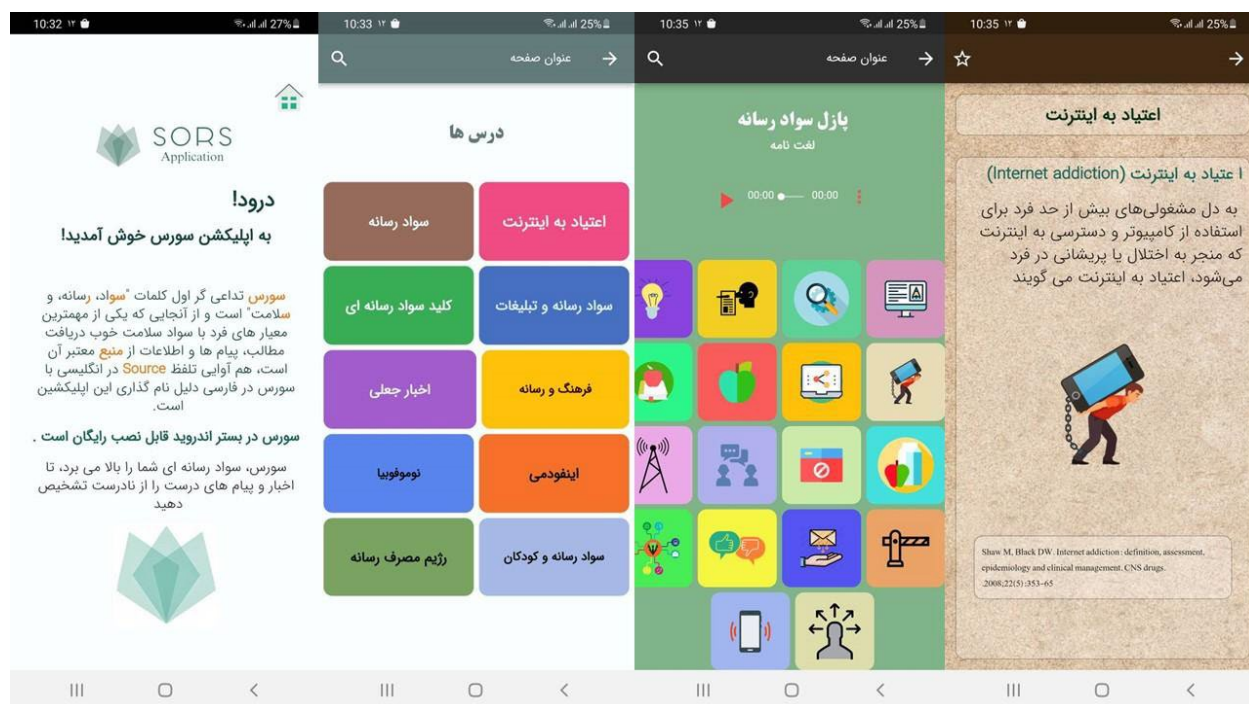

Supplement: Supplementary file 1 — contains sections of the SORS-App. [file hpp-13-227-s001.pdf]
